# Mechanical Performance and Failure Analysis of a 3D-Printed “Continuous Layer–Lattice Layer–Continuous Layer” Sandwich Structure

**DOI:** 10.3390/polym15214283

**Published:** 2023-10-31

**Authors:** Daming Nie, Lingyu Kong, Yu Zhang, Xingyu Qiu, Yili Fu, Jason Gu

**Affiliations:** 1 Research Center for Intelligent Robotics, Zhejiang Lab, Hangzhou 311100, China; niedaming1989@163.com (D.N.); kongly@zhejianglab.com (L.K.); fuyl@zhejianglab.com (Y.F.); 2 School of Mechanical and Energy Engineering, Zhejiang University of Science and Technology, Hangzhou 311100, China; qxyuust@gmail.com; 3 Department of Electrical and Computer Engineering, Dalhousie University, Halifax, NS B3M 1A2, Canada; jgu@zhejianglab.com

**Keywords:** carbon fiber, fiber reinforced material, sandwich structure, lattice layer

## Abstract

Sandwich structures are engineered with continuous layers surrounding the inner lattices, which combines the advantages of the high strength of the continuous layer and the light weight of the lattice layer. They are widely employed in weight-critical energy-absorbing engineering fields such as aerospace, automobile, and robotics. However, the application of sandwich structures made of polymer matrix composites is still limited due to lack of essential performance investigation and adequate reference data. The following innovative works are accomplished in this paper: (i) Continuous long glass fiber (CGF) is employed within the continuous layer of the sandwich structure, with composite short carbon fiber/polyamide (SCF/N) applied within the lattice layer. (ii) Sandwich structures with different cell types and orientations of the lattice infills are designed and prepared by additive manufacturing. (iii) The basic mechanical properties of the sandwich structures, i.e., the bi-directional tension/compression compound performance, failure modes and mechanisms in characteristic directions, are analyzed systematically. (iv) The effects of geometric features on the three-point bending properties of L-shaped sandwich structures are investigated and compared with those of pure SCF/N structures. The results show that the bending resistance per unit weight was up to 54.3% larger than that of pure SCF/N, while the weight could be decreased by 49%, and the bending flexibility before fracture could be increased by 44%. These studies contribute fundamental research data to the application of sandwich structures prepared by fiber reinforced polymer matrix composites.

## 1. Introduction

The sandwich structure with the form “continuous layer–lattice layer–continuous layer” is widely employed as a light-weight solution in aerospace [1,2], automobile [3,4] and aerodynamic structures [5,6], as well as in energy absorption components [7,8], because of its large structural stiffness and small equivalent density. In particular, the emergence of additive manufacturing technology brings the potential of manufacturing complex sandwich structures for broader industrial needs that cannot be achieved by traditional machining [9]. For example, Kang et al. [10] fabricated curved sandwich structures with a 3D lattice core and proposed a pinned method to improve the adaptability between the core and the skins. Rajpal et al. [11] analyzed the natural frequency and damping ratio of sandwich structures with various orientations of core layers made by additive manufacturing. Pierre et al. [12] produced sandwich structures with complex geometric shapes through 3D printing technology, which provides them with wider sound absorption performance than traditional honeycomb cell sandwich panels.

Among the parent materials of sandwich structures, high strength alloy is generally used for large bearing and high temperature service capacity. Du et al. [1] prepared a sandwich structure with two layers of corrugated core plates made of Ti_2_AlNb alloy, which could reduce the weight of devices serving at high temperature. Resin materials are generally employed under low load-bearing and normal temperature service conditions. For example, Serra et al. [13] used PLA to fabricate scaffolds that possess adequate pore size and inter-connectivity in accordance with the injured tissue. Ji et al. [14] prepared two types of aggregation-induced emission (AIE) stereo-lithography resins to manufacture a three-dimensional lattice structure. Compared with pure resin, fiber reinforced resin matrix composites (CFRC) [15] show better comprehensive properties in some application scenarios [16,17]; long continuous carbon fiber (CCF) reinforced PLA composite with 6.6% volume fraction of fibers exhibited a 599% and 435% higher elastic modulus and tensile strength, respectively, compared with pure PLA in the fiber direction. Karas et al. [18] selectively printed adhesive and polymer powder on discontinuous carbon fibers and then compressed, heated and post-processed the material to form lattice parts. The results show that for the 15% volume fraction of short fibers, the tensile strength can reach 97 MPa, and the elastic modulus is 8.9 GPa.

We take an innovative approach, applying the continuous fiber as the continuous layer material of the sandwich structure and using the short carbon fiber reinforced polymer as the material of the lattice layer. The long continuous fibers are laid in the specified directions by 3D printing [19]. High-strength fibers along the ply direction significantly improve the tensile and shear forces of composite structures [20,21]. Additionally, the short fiber reinforced composites used to fill the lattice layer can be periodically arranged with higher flexibility in forming the complex shape of the lattice layer [22]. Therefore, such material collocation can not only better meet the requirements of strength and weight reduction in theory, but also obtain sufficient structural stiffness through the flexible design of the lattice layer.

In this paper, firstly, the sandwich structures are prepared by additive manufacturing, and the continuous long glass fiber is employed within the continuous layer, while the composite short carbon fiber/polyamide is applied in the lattice layer. Secondly, the essential mechanical properties of the sandwich structures, i.e., the uniaxial tensile and bi-directional tension/compression compound performance, failure modes, and mechanisms in characteristic directions, are analyzed systematically. Thirdly, the effects of geometric features on the three-point bending properties of L-shaped sandwich structures are investigated and compared with those of pure polyamide properties. The results show that the bending resistance per unit weight was 54.3% larger than that of pure SCF/N and the bending flexibility before fracture increased by 44%. These studies serve as a fundamental research reference and provide systematic property data for material selection and assessing the mechanical characteristics of resin matrix composite sandwich structures.

## 2. Materials and Methods

### 2.1. Material Selection

**Figure 1:**
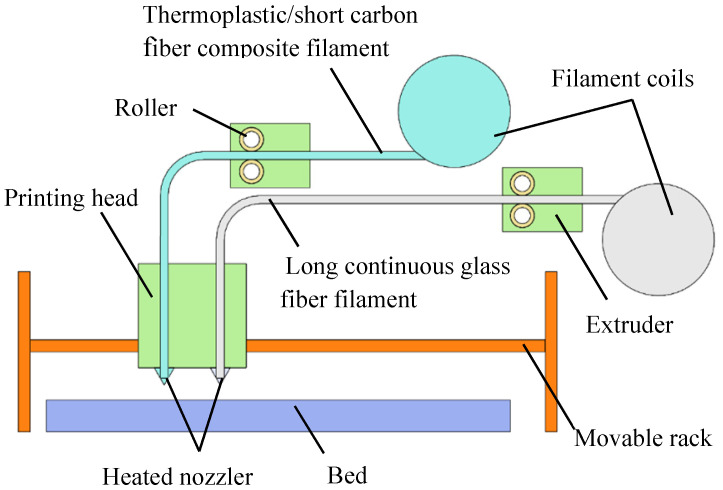
Schematic diagram of printing equipment.

The materials in these experiments included continuous glass fiber (CGF) and a compound of short carbon fiber/polyamide (SCF/N). As the material of the continuous layer, the long continuous glass fiber is made of high-strength fiber filaments whose surface is evenly coated with adhesive and twisted into fiber coils (Figure 1). The diameter of the continuous glass filament is 0.35 mm, and the average surface roughness is Ra1.6. When applied, the fiber filament is delivered to the nozzle and the polymer on the surface is heated into a molten state in real time through the conveying device, which consists of filament coils, rollers and printing heads.

The printing of the designed models was completed using the control program to plan the movement path of the printing head and set parameters. The material of lattice layer was a compound of short carbon fibers and polyamide. The short fibers and molten polyamide were mixed without pressure to form a non-oriented compound. The volume fraction of the fiber was 35%, at which ratio the substrate could reach the best comprehensive performance. The surface of the short fiber was modified to improve the interface strength. The printing machine employed was the Mark 2 from the company Markforged (Boston, MA, USA).

### 2.2. Sample Preparation

In order to analyze the basic mechanical properties of the proposed sandwich structure, two types of samples were prepared in this experiment. The first was a long strip sample used for uniaxial tension and bi-directional tension/compression deformation; the other was an L-shaped specimen for three-point bending tests. As for the strip samples, three groups of samples were prepared to analyze the mechanical properties of different materials (Table 1). The first group was prepared for the compound of short carbon fiber and polyamide (SCF/N). In order to eliminate the anisotropy caused by the scanning path, the orientation of each layer of the sample was set as 45°/135°/45° sequentially.

**Figure 2:**
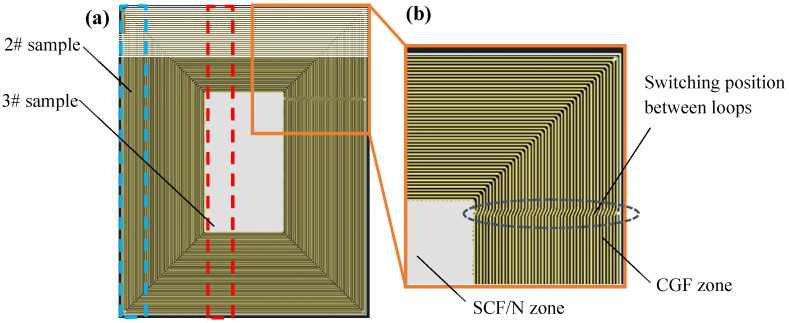
Schematic diagram of manufacturing process of sample 2# and sample 3#: (**a**) complete morphology; (**b**) enlarged view of inter-loop transition zone.

The 2# sample was made of continuous glass fiber (CGF). It is difficult to prepare a sample whose whole filaments are strictly parallel to the length direction due to the limitation of the machine program. Therefore, as an alternative, a plate (Figure 2) with the dimensions 150.0 mm × 120.0 mm × 2.0 mm was designed and the sample was cut from the marked position (blue dotted line) in Figure 2, i.e., along the edge zones in the width direction, similar to the technique applied in [22].

Although the fibers near the two ends of the sample were not parallel to the length direction, these positions were held by clamps during uniaxial tension; therefore, the impact of errors in modeling on the tensile performance of the sample was very small. The designed plate shown in Figure 2a is 100% CGF on the periphery, and 100% SCF/N at the core. The length ratio between the two ends and the core is close to 1:1. The transition zone of the scanning loops is fixed in the length direction of the plate (Figure 2b).

**Figure 3:**
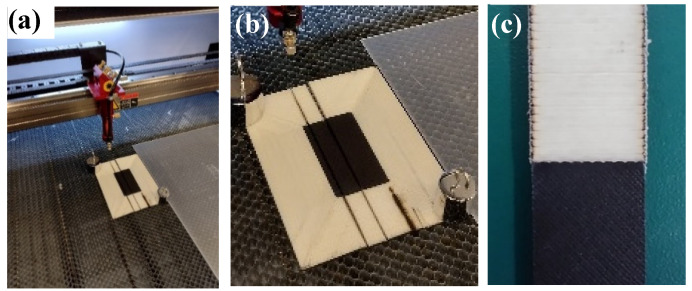
CO_2_ gas cutting machine employed to prepare samples and surface morphology of samples: (**a**) CO_2_ gas cutting machine; (**b**) samples preparation; and (**c**) surface morphology of samples.

The 3# sample is made of 50% SCF/N and 50% CGF, which was adopted to test the weakest strength among the CGF/CGF interface, CGF/(SCF/N) interface and SCF/N. Sample 3# was taken from the center zone (red dotted line) of the plate in Figure 2. Taking into account that the plate is thin and contains glass fiber, a CO_2_ cutting machine was employed (Figure 3a) to prepare the sample after a failed attempt using laser cutting, which caused the sections to burn (Figure 3b, burn mark at the lower right zone of the sample). Clamping was required during cutting because the thin plate is inevitably warped during forming. The cutting power of the machine was set to 6 W and the speed was 10 mm/s, and with these settings, a smooth incision was obtained (Figure 3c).

**Figure 4:**
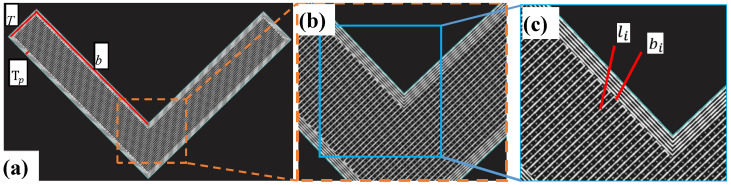
Schematic diagrams of density calculation in lattice layer: (**a**) Scanning path of sample section; (**b**) partial enlarged view; and (**c**) secondary enlarged diagram.

Instead of simple shape like a plate or bar that only needs to undergo uniaxial tension or compression, sandwich structural parts are often made into complex models and bear bending/torsion forces in three orthogonal directions in actual industrial applications. In this experiment, the commonly used L-shaped parts were designed to analyze the effects of the characteristics of this structure on performance. The L-shaped parts were symmetrical from left to right, and the overall dimensions were 135.8 mm × 81.3 mm × 10.0 mm (Figure 4a,b). The L-shaped parts consist of a continuous layer on the outside and a lattice layer at the core. The unilateral thickness of the continuous layer was 2 mm, and the thickness of the lattice layer was 15 mm. The materials of the continuous and lattice layers are CGF and SCF/N, respectively.

The specific geometrical parameters of the lattice layer in the L-shaped parts are illustrated in Table 2. The four mostly used types of cells, which are triangle, rectangle, hexagon and gyroid [23], are represented in the samples from 4# to 7#, respectively. The volume fractions and orientation are all the same and equal to 35% and 0° for convenience of comparison.

It is difficult to calculate the force under complex service conditions through the formulas in structural mechanics directly [24]. The finite element calculation is theoretically feasible but limited by its great demand for computational power. Recently, the equivalent element method (EVM) was proposed to reduce the amount of calculation [25]. However, this method requires the reliable input of material property parameters; thus, the most reliable and fastest method is still experimental testing.

The orientation of cells in the lattice layer is also a crucial factor in designing a sandwich structure. The lattice layer is formed by the periodic arrangement of cells, not by the non-oriented combination of metal poly-crystals [26], thus representing strong anisotropy. Five groups of L-shaped samples were prepared in this experiment, with the lattice orientations 0°, 19°, 38°, 57° and 76°, respectively (Table 3). The ideal orientation settings are 0°, 22.5°, 45°, 67.5° and 90° but could not be realized by the printing machine. The number of cells in the lattice layer varies depending on the different orientations. In order to eliminate the effect of material occupancy, the cell calculation method is proposed to determine the width of the lines in lattice layer. The formula is:

(1) $$\varphi =\frac{\sum (l_{i}+b_{i})\times T}{A}$$

where *φ* is the volume fraction of material in lattice layer, $l_{i}$ is the total line length of the lattice layer, $b_{i}$ is the increased line length in the lattice layer to keep the path continuous (Figure 4c), *T* is the width of the line, and *A* is the area of the lattice layer. This formula is similar to that applied in [27], with both following the basic principle of conservation of matter.

The above calculations were executed by the program automatically. The side length of the lattice cell and the scanning path of the nozzles were automatically determined by the program according to the diameter of the employed glass and polyamide fibers for good production quality. Subsequently, the total length of the scanning line in the lattice layer was determined, and finally, the line width was calculated according to the line length and the required material volume fraction.

The Instron 6800 universal testing machine was applied to conduct the three-point bending test of the L-shaped parts. The compression speed was 6 mm/min. Graphite powder was sprinkled on the table of the testing machine, which allows sliding of the lower end of the L-shaped parts, preventing instability due to static friction in compression.

### 2.3. Accurancy Measurement of Samples

**Figure 5:**
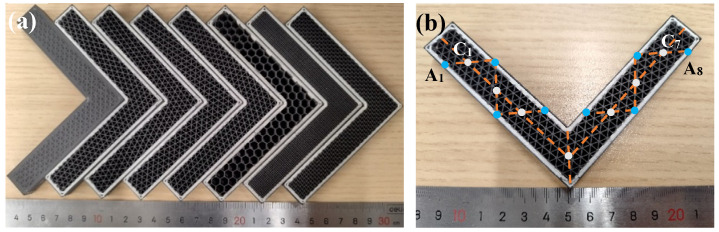
Collection of samples with different cell types and orientations: (**a**) collection of samples; and (**b**) positions of measure points.

Figure 5a shows the samples successfully processed based on the parameters designed above. The surface of the L-shaped parts are neat without holes, slag, cracks or other defects. The fiber filament is laid with accurate positioning and is of good quality. The maximum error of the feature sizes (thickness of continuous layer, width of the lattice line) was measured by a vernier caliper whose minimum graduation is 0.001 mm. The measure points were selected to be equally distributed along the L-shaped contour, and the positions were symmetrical from left to right (Figure 5b).

## 3. Experimental Results

### 3.1. Analysis of Interface Strength

**Figure 6:**
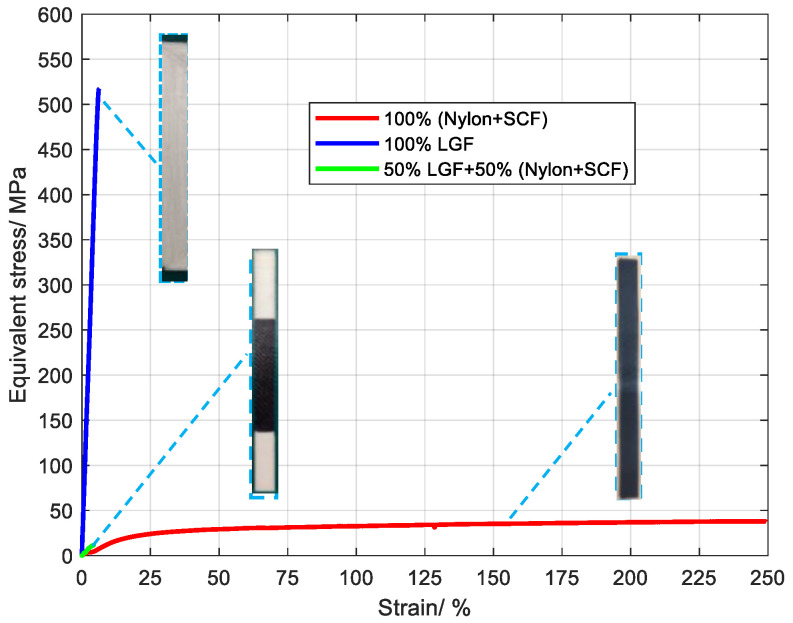
Equivalent stress–strain curves of three materials under uniaxial tension.

**Figure 7:**
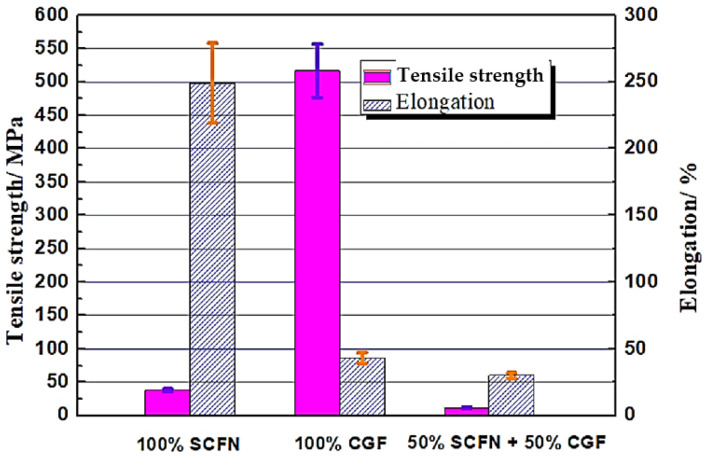
Elongation and tensile strength of three materials.

The stress–strain curves of three samples are shown in Figure 6, in which the red curve represents 100% SCF/N, the blue curve represents 100% SCF, and the green curve represents 50% CGF and 50% SCF/N. The elongation of 100% SCF/N reached 250%, indicating good hyperelasticity, but the tensile strength was 94.1 MPa (Figure 7), which is higher than the 100% polyamide [28]. This results from the restriction of carbon fiber to polyamide in deformation. The tensile strength of 100% CGF was 520 MPa, which results from the high strength of glass fiber in the length direction. This performance was higher than that of SS 304 steel [29], and there was no obvious anisotropy.

The tensile strength was less than 100% (Polyamide + SCF) for the sample with 50% CGF and 50% SCF/N, and the elongation was much smaller than the latter (Figure 7). This may occur for two reasons. One is that the interface strength between 100% LGF and 100% SCF/N is small, and the other is that the long glass fibers are distributed perpendicularly to the direction of tensile force, and the adhesion between the glass fibers is weaker than the interface strength between 100% (Polyamide + SCF) and 50% (CGF + 50% SCF/N) materials. The fracture position of the sample is within the zone of CGF, indicating that CGF is the cause.

### 3.2. Failure Analysis of Samples

#### 3.2.1. Failure Analysis of SCF/N Sample

**Figure 8:**
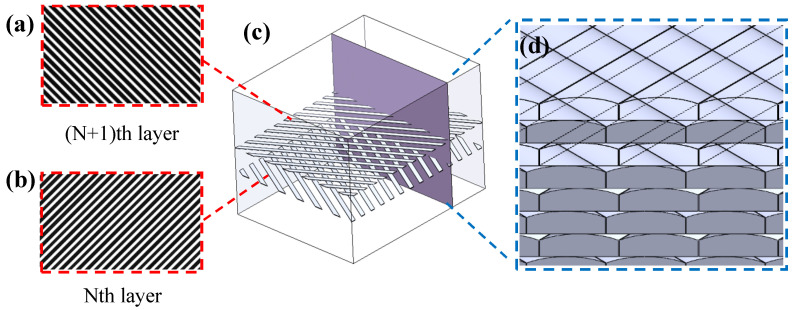
Schematic diagram of the selected section position for analysis: (**a**) scanning track of Nth layer; (**b**) scanning track of (N + 1)th layer; (**c**) schematic diagram of section position and (**d**) schematic diagram of track on section.

**Figure 9:**
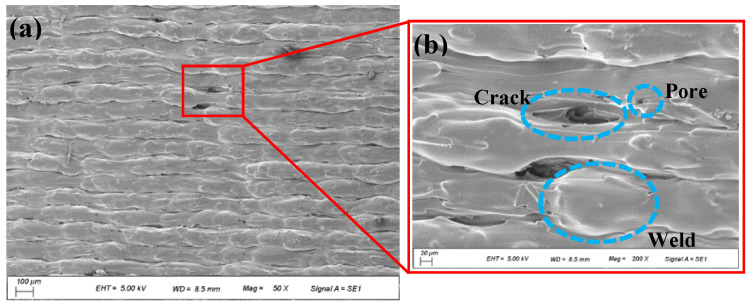
Morphology of section: (**a**) morphology; and (**b**) partial enlarged view.

The scanning track of each layer of the 100% SCF/N sample is illustrated in Figure 8. The layers are arranged in alternate orientations, i.e., if the scanning track is laid at 45° in Nth layer, then the orientation in the (N + 1)th layer is 135°, which is similar to [30]. There are obvious delaminations on the cross-section of 0°, including long and narrow cracks between interlayers, whose length reached more than 100 μm, and the height exceeds 20 μm (Figure 9a,b). A similar phenomenon was reported in literature [31]. There are three possible causes for the defects. The first is the long time interval between two neighbouring layers, which results in the temperature of the lower layer being much lower than that in the upper layer that is forming. Therefore, the temperature gradient is large during interlayer fusion. The second cause is the reduced volume of the molten polyamide during cooling. The third cause is the surface tension of molten SCF/N itself; short carbon fibers increase the surface tension of polyamide, leading to worse interlayer fusion.

Although the intra-layer material is also filled by fusion of inter-tracks, the time interval is relatively short; thus, the fusion is more sufficient. Light defects still exist in the form of pores whose diameter is ~2 μm locally. The reason is also the reduced volume of polyamide during cooling. Another defect is the uneven thickness of layers. Weld beads can be observed (Figure 9b) at the exact scanning position of the nozzles. Short carbon fibers are distributed inside the layers without specific orientation, while no fibers appear at the interfaces between layers.

**Figure 10:**
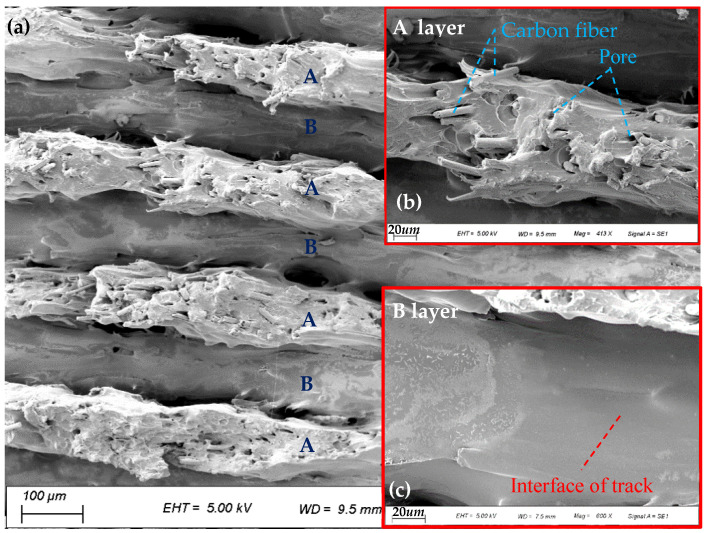
Tensile fracture morphology of SCF/N sample: (**a**) tensile fracture of sample; (**b**) enlarged view of layer A; and (**c**) enlarged view of layer B.

The fracture morphology of the prepared 100% SCF/N sample in uniaxial tension is shown in Figure 10a. The two obvious characteristic layers A and B are distributed alternately. Layer A is relatively thinner and includes a quantity of holes caused by the absence of the pulled out short fibers and the exposed fiber filaments (Figure 10b), which is also reported in [32]. The orientation of the short fibers tends to be consistent, parallel to the tension direction. The interface between the fiber filaments and the polyamide matrix is closely connected. The thickness decreases about two-fold compared with the B layer, indicating that the matrix material possesses good elongation. Layer B is flat with greater thickness (Figure 10c), and carbon fibers cannot be observed. These results indicate that the fracture position of the B layer is the interface of tracks inside the layer.

In order to analyze the width shrinkage of this material before fracture, the parameter δ is proposed as follows:

(2) $$\delta =\frac{(T_{B}-T_{A})\times L_{0}}{T_{B}\times \left(L-L_{0}\right)}\times 100\%$$

where $T_{A}$ and $T_{B}$ are the thickness of Layer A and B, respectively. $L_{0}$ and L are the original length and dynamic length in tension of the sample.

**Figure 11:**
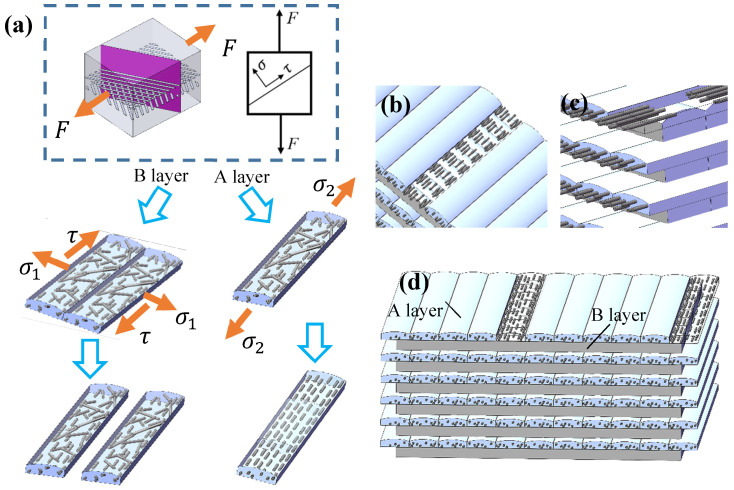
Schematic diagram of orientation variation in SCF/N in tension: (**a**) schematic diagram of track fracture with two orientations; (**b**) short fiber orientation; (**c**) fracture diagram; and (**d**) schematic diagram of sandwich sample.

On the coupling action of shear force τ parallel to the inter-track and the tensile stress $\sigma_{1}$ [33,34], which is perpendicular to the tracks as illustrated in Figure 11a, Layer B breaks before layer A. The fracture is mainly shear deformation accompanied by weak tensile deformation. As for layer A, the tensile stress $\sigma_{2}$ increases to nearly twice the original value after the fracture of layer B. Under the action of tensile force, the relatively weakly oriented A layer [35] becomes thinner and the short fibers tend to be parallel to the drawing direction, resulting in the fracture morphology shown in Figure 11b,c.

(3) $$\tau \approx \sigma_{1}=\frac{\sqrt{2}\lambda_{1}F}{2\times \sum (T_{A}+T_{B})\times W_{1}}$$

(4) $$\sigma_{2}=\frac{\sqrt{2}\lambda_{2}F}{\sum T_{A}\times W_{2}}$$

where *F* is the tensile force of the sample, and $W_{1}$ and $W_{2}$ are the width of the sample in different tensile stages. $\sum T_{A}$ and $\sum (T_{A}+T_{B})$ are the total thickness of Layer A and Layer A plus B, respectively. $\lambda_{1}$ and $\lambda_{2}$ are the parameters representing the orientation variation of each layer in tension.

**Figure 12:**
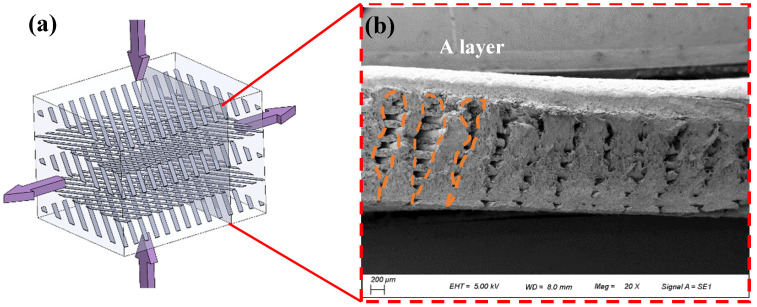
Variation in layer characteristics during tension–compression composite deformation: (**a**) schematic diagram of tensile and compressive forces; and (**b**) fracture morphology.

Tension and compression compound deformation tests were performed on the samples to obtain conditions closer to the real service condition. The 0° cross-section is shown in Figure 12a. Two characteristics can be observed in the section (Figure 12b): (1) Inter-track crack inside layer. Cracks are formed due to elastic recovery after unloading because of the inter-track separation during compression. The cracks on upper position are larger than those below due to the influence of lattice displacement. (2) The interlayer separation is oriented. The separation occurs in the direction of 45° from the width direction of the sample, while the layer in the direction of 135° has no effect. The reason is that the 45° oriented layer breaks at a small elongation, and the layer thickness is greater. However, the 135° direction layer becomes less thick, and there is an obvious interlayer gap after springback due to the large elongation.

### 3.3. Performance of L-Shaped Sandwich Structure

**Figure 13:**
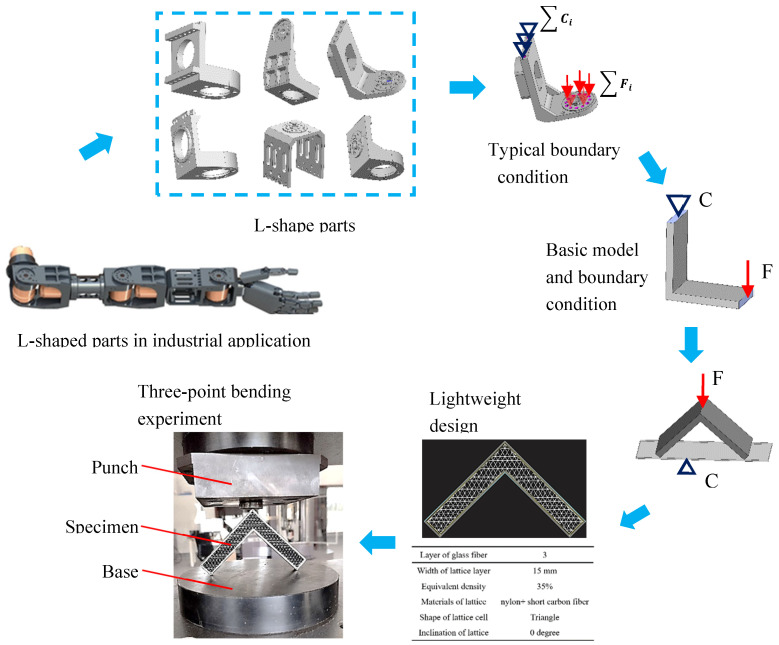
L-shaped sandwich structural samples and three-point bending test based on application requirements.

Fiber reinforced composites are mostly used in industry as panels or bars, and the introduction of additive manufacturing technology makes it possible to form complex structural parts. The strength of fiber reinforced materials is generally lower than that of metal, so they can be used as lightweight structural parts in mechanical systems with relatively small loads, such as arm structural parts of robots. As shown in Figure 13, 70% of the structural parts in the humanoid robot arms are L-shaped, and the specific strength of fiber reinforced composite is much larger than 6061 aluminum alloy, making it ideal material for robotic arms.

The deformation properties and failure modes of L-shaped parts cannot be calculated from standard tensile samples. It is necessary to carry out basic performance tests. Figure 13 illustrates the process of determining the performance test procedures of L-shaped parts. First, the general loading form of L-shaped parts is analyzed, such as the $\sum C_{i}$ and load conditions $\sum F_{i}$. Second, the model and loading conditions are simplified to form constraint condition C and loading condition F. Considering the operability of the testing machine, the loading form of the L-shaped specimen is further simplified. The two ends of the L-shaped sample are placed the on the platform and a downward compression force is applied at the middle point. Thus, the sample has horizontal translation freedom at the two edge ends. Subsequently, the structural parameters of the L-shaped parts are determined, and the tests for three-point bending experiment are conducted.

**Figure 14:**
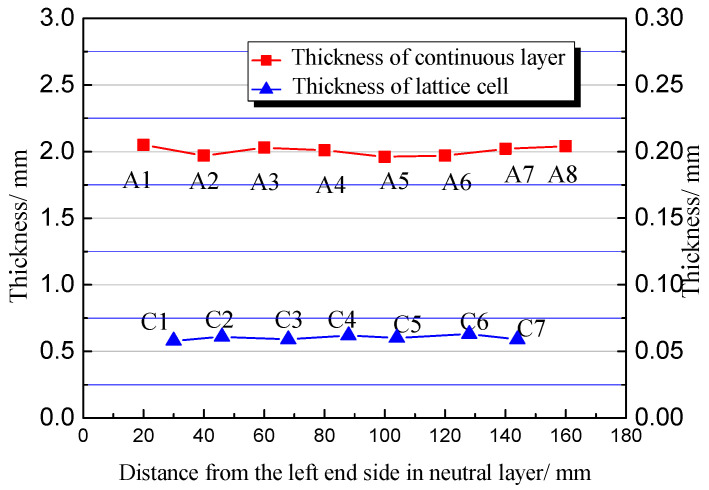
Thickness variation of continuous layer and lattice layer of the sample. The lattice cell is a triangle oriented at 0°.

Before mechanical testing of the specimen, it is necessary to determine its forming quality. In this experiment, the triangular lattice with 0° orientation is taken as an example. The points illustrated in Figure 5b are used to detect the thickness uniformity. The results show that the maximum thickness difference of the continuous layer (Figure 14) is 91 μm, and the thickness difference of the lattice layer is 4 μm, indicating that the thickness of each layer is consistent under the unified control of the program of the printing machine.

### 3.4. Effect of Cell Type on Structural Mechanical Properties

**Figure 15:**
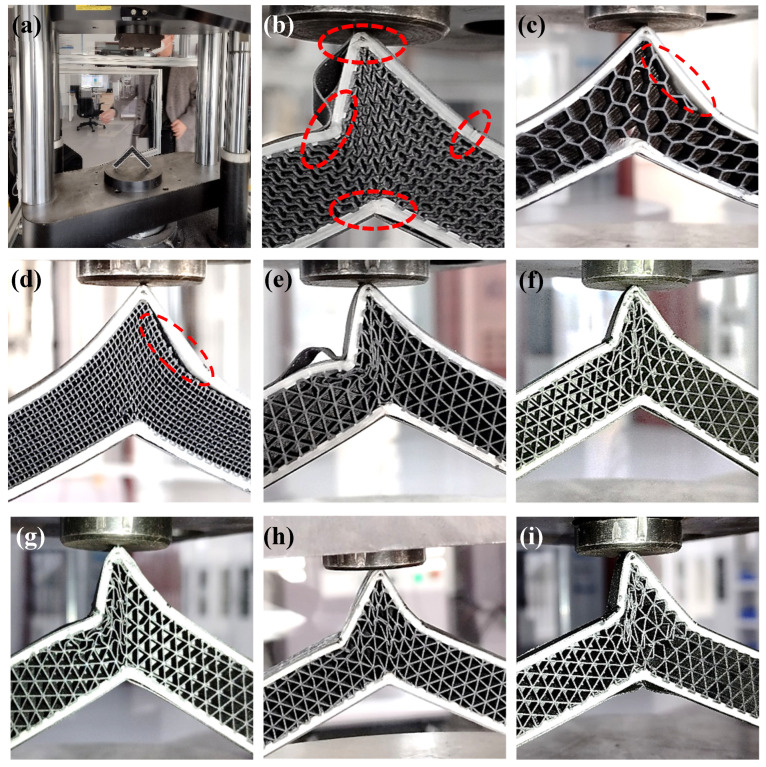
Failure of sandwich structure with various cell types and orientations during three-point bending deformation: (**a**) sample placement; (**b**) compression of gyroid-type sample; (**c**) compression of hexagon-type sample; (**d**) compression of rectangle-type sample; (**e**) compression of 0° oriented triangle-type sample; (**f**) compression of 38° oriented triangle-type sample; (**g**) compression of 59° oriented triangle-type sample; (**h**) compression of 76° oriented triangle-type sample; and (**i**) compression of 76° oriented triangle-type sample.

According to the failure mode analysis, when the sample with triangular cells is bent and fractured, it shows several characteristics: (1) The external angle decreases with deformation (Figure 15b). (2) The internal angle increases with deformation. (3) The continuous fiber layer breaks near the corner. (4) Obvious deformation is produced in the lattice layer near the corner. (5) The deformation near the corner on both sides is asymmetric because the formation conditions on both sides are not completely the same. For example, the inter-loop switching position (Figure 2b) between layers is on the left side.

The reason for the low peak value of hexagonal and rectangular cells is that the interface between the lattice layer and the continuous fiber layer is completely stripped (Figure 15c,d). Thus, the boundary constraints of the lattice layer are reduced. The outer layer of the fiber layer does not bend, so the peak value of the bending force is small. Although the lattice layer underwent significant deformation, the deformation resistance caused by this deformation is small.

**Figure 16:**
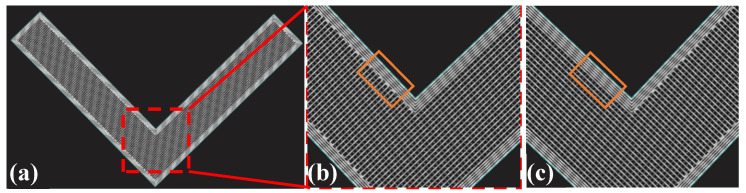
Lattice laying method of samples with rectangular cell type: (**a**) scanning track on section; (**b**) partial enlarged view of the nth floor; and (**c**) partial enlarged view of the (N + 1)th floor.

As for the same equivalent volume fraction of materials, the bending forces produced by different configurations vary clearly. Comparing their geometric characteristics, the concept of shape factor *ψ* is proposed in this paper to qualitatively characterize the bending resistance of an L-shaped sample.

(5) $$\psi =\Gamma \left(b_{i},∆,\theta \right)$$

where $b_{i}$ denotes the length of single lap unit, as illustrated in Figure 5. The smaller the value of $b_{i}$, the greater the bending force. ∆ denotes the total lap area as the overlapping position of each lattice layer is not necessarily the same. Considering, for example, the rectangular cell sandwich structure, the actual overlapping area is reduced due at the same position to the existence of interlayer overlapping (Figure 16), which also causes peeling during bending. θ is the maximum included angle of the lattice cell. The higher the value of θ, the smaller the peak compression force.

It is difficult to obtain the complete expression of the shape factor *ψ* due to insufficient experimental support. However, it can be determined that the above factors are the main ones and effective for qualitative analysis of the bending resistance of the sandwich structure.

The three-point bending stress σ for full continuum plates in three-point bending can be determined according to the following formula:

(6) σ=MxIzy =Fl2bh312y= 6Flbh3 y

where *b* and *h* are the width and thickness of the specimen, respectively, l is the bending span, *F* is the bending force, $I_{z}$ is the moment of the bending inertia, $M_{x}$ is the bending moment, and *y* is the displacement of the bending. For V-shaped parts, the bending span l also changes during compression:

(7) l=f(y,E,μ,A)

where *E* is the equivalent elastic modulus, *μ* is the sliding friction coefficient of the sample, and *A* is the contact area between the samples and the base. For a sandwich structure, the bending moment of the continuous layer is calculated consistent with the ideal continuum plate:

(8) $$I_{z1}=\int_{A}^{}y^{2}dA=\frac{W\left(T^{3}-T_{C}^{3}\right)}{12}$$

where $I_{z1}$ is the bending moment of inertia of the two continuous layers of the sandwich structure, *A* is the differential area of stress, *y* is the distance of the differential unit from the neutral layer of bending, *W* and *T* are the width and thickness of the sandwich structure, and *T_C_* is the thickness of the lattice layer.

For the lattice layer,

(9) $$I_{z2}=\int_{A}^{}y^{2}dA=\frac{W}{12}{\tilde{T}}^{3}$$

where $I_{z2}$ is the bending moment of inertia of the lattice layer, $\tilde{T}$ is the modified thickness on the basis of the lattice layer *T*, whose value is affected by the type and side length of the lattice, material volume fraction, lattice orientation, interface strength between the lattice layer and continuous layer, etc.

In view of the above analysis, the equivalent bending stress σ of a sandwich structure cannot be directly calculated by theoretical or empirical formulas due to the influence of geometry, force and engineering conditions. Therefore, the compression force–displacement curve is used to study the influence of element type.

**Figure 17:**
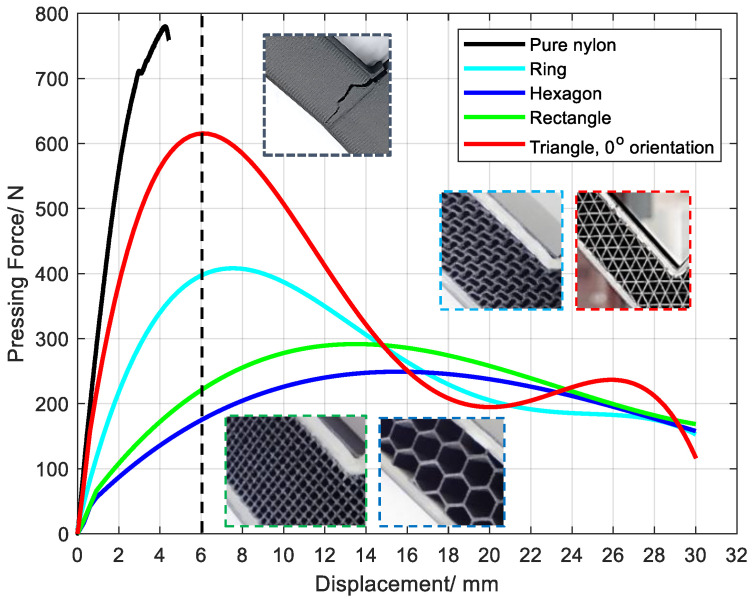
Effect of compression force on compression stroke for samples with various cell types.

Although the material equivalent density of the lattice layer is set at 35% for sandwich structures with various cell types, the force–displacement curve during bending deformation is quite different, which is similar to [11]. For the structures occupying the four types of lattice cells with an orientation of 0°, the following common characteristics are possessed: (1) The trend is the same, with an increase in deformation first and then a decrease (Figure 17). (2) The compression force and displacement show a relationship of quartic polynomial, and the second wave peak of the triangle is the error introduced by the function fitting. There are also several differences: (1) The compression stroke at the peak is different, which is inversely proportional to the time of its arrival. (2) The variation ratio of the compression force decreases with the peak value of compression.

The peak compressive force of the pure polyamide specimen was 780 N, the compression before breaking was 4.2 mm (Figure 17), and the weight was 36.81 g. For the 0° triangle specimen, the compression amount was 6.37 mm, the peak compression force was 614 MPa, the weight was 18.77 g, and the compression before breaking was 6.06 mm. Similar to the concept of specific strength, the ratio of compression force to weight is used to measure the bending resistance of L-shaped parts, and the compression amount at the peak of compression force is employed to measure the bending compliance of the L-shaped part. The “similar specific strength” of pure polyamide was 21.189 N/g, and the “similar specific strength” of the 0° triangular element specimen was 32.71 N/g, which is 54.3% larger than that of pure SCF/N. The compression at the peak of compression force increased by 44%.

The quantitative relationship between compression force and displacement can be obtained by data fitting. The error of the quartic polynomial relation is smaller in this experiment, and the fitting parameters and errors of different groups are listed in Table 4.

(10) $$y=ax^{4}+bx^{3}+cx^{2}+dx+e$$

### 3.5. Effect of Lattice Orientation on Structural Mechanical Properties

**Figure 18:**
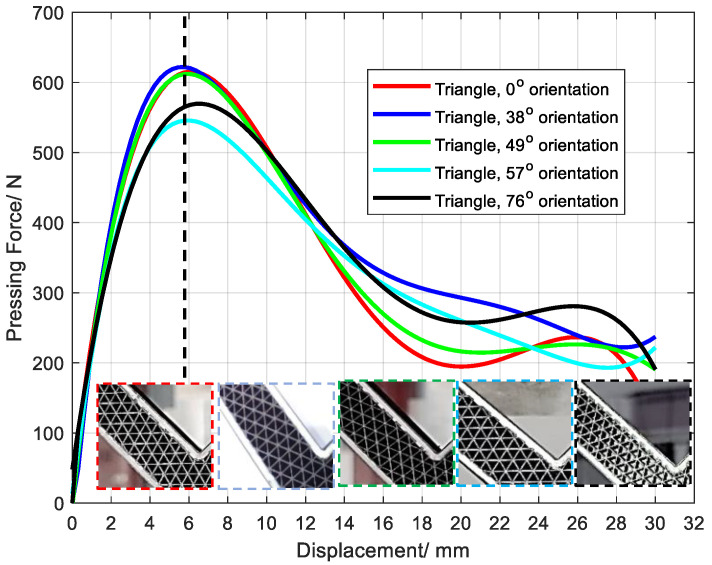
Effects of compression force on stroke for samples with various lattice orientations.

The orientation of the lattice also could significantly affect the deformation performance of the lattice layer [36]. For triangular lattice layers with different orientations in this paper, their peak compression values in the three-point bending tests were not the same (Figure 18). Also, the amount of compression required to reach the peak value, and the deformation and failure forms were not consistent. Previous analysis showed that the SCF/N structure is obviously anisotropic. However, the effect of orientation in the L-shaped structure is small, which results from the continuous layer of glass fiber in the outer layer and the deformation of three-point bending. This phenomenon provides a solution to avoid the influence of anisotropy on the mechanical properties of the sandwich structure.

## 4. Conclusions

In this paper, the materials of three characteristic layers of a sandwich structure were innovatively designed and prepared by additive manufacturing. The failure modes and mechanisms of the tested specimens were analyzed, and the three-point bending performance were systematically investigated. The following conclusions can be drawn:

(1) The mechanical properties and failure modes of carbon fiber reinforced polyamide materials exhibit evident anisotropy in deformation by biaxial tension/compression. The cause is different scanning paths during 3D printing, and the differences cannot be eliminated through the fusion of polyamide, mainly due to temperature variation in processing and the surface tension of hot-melt polyamide.
(2) Two kinds of fracture forms exist simultaneously in the 100% SCF/N under uniaxial tension. One is the shearing off, mainly by the action of shear force. The fracture section is flat and the layer thickness is nearly unchanged. The other is cracks that form under tensile force. The fiber orientation in the layer is approximately parallel to the tensile direction, and the layer thickness is significantly reduced. Parameter δ proposed in Section 3.2.1 could characterize the width shrinkage of this material before fracture.
(3) Fractures along the direction of continuous filament are similar to the “cleavage fracture” in poly-crystal metal, which leads to two parallel disconnected surfaces. These fractures are mainly caused by colloid stripping on the surface of the filament, supplemented by inner tearing of the colloid, and accompanied by fiber shearing in about 10% of the area of the cross-sections.
(4) The concept of shape factor is proposed to evaluate the three-point bending resistance by analyzing the failure characteristics and causes of the L-shaped specimens. The parameters of total lap area, the overlapping position of each lattice layer and the maximum included angle of the lattice cell are the crucial factors.
(5) The bending resistance per unit weight of the L-shaped part was 54.3% larger than that of pure SCF/N, while the weight was decreased by 49%. The bending flexibility before fracture increased by 44%. This lightweight effect has obvious application value in some scenarios in which it is necessary to maintain the appearance, shape and a certain degree of stiffness of the workpiece, while also remaining sufficiently lightweight, such as in the upper arms of robots.
(6) After formula derivation, the bending strength of the L-shaped specimens could not be directly calculated by the formulas. The relationship between compression force and displacement of L-shaped parts is a quartic polynomial. The force–stroke curves of L-shaped specimens with different orientations varies little, and the design of the sandwich structure can significantly reduce the effect of lattice anisotropy, which could provide a method to reduce the anisotropy of composite materials in use.

## Figures and Tables

**Table 1:** Materials and orientations of various samples.

Sequence Number	Composition of Materials	Layout Design
1#	Short carbon fiber + Polyamide (SCF/N)	45°/135°/45° from the length direction
2#	Continuous glass fiber (CGF)	Parallel to the length direction
3#	50% CGF + 50% (SCF/N)	CGF on the two ends, SCF/N in the middle

**Table 2:** Samples of different cell types prepared in the experiment.

Sequence Number	Proportion of Volume in Lattice Layer (%)	Cell Type	Orientation (Degree)	Contour Shape
4#	35	Triangle	0	L shape
5#	35	Rectangle	0	L shape
6#	35	Hexagon	0	L shape
7#	35	Gyroid	0	L shape

**Table 3:** Specimens with different orientations.

Sequence Number	Proportion of Volume in Lattice Layer (%)	Cell Type	Orientation (Degree)	Contour Shape
4#	35	Triangle	0	L shape
8#	35	Triangle	19	L shape
9#	35	Triangle	38	L shape
10#	35	Triangle	57	L shape
11#	35	Triangle	76	L shape

**Table 4:** Fitting parameters and errors of different lattice layers.

	Ring	Hexagon	Rectangle	0° Oriented Triangle	38° Oriented Triangle	49° Oriented Triangle	57° Oriented Triangle	76° Oriented Triangle
a	−0.0069	−0.0004	−0.0003	−0.0186	−0.0155	−0.0166	−0.0109	−0.0144
b	0.527	0.0404	0.0529	1.2864	1.0856	1.1709	0.7962	1.0131
c	−13.8	−1.9572	−2.815	−29.626	−25.439	−27.478	−19.552	−23.87
d	129.95	36.985	49.506	235.16	209.44	222.59	168.29	198.44
e	9.1793	16.223	15.275	18.288	51.546	28.975	72.528	37.411
$R^{2}$	0.967	0.998	0.9985	0.997	0.9627	0.994	0.9734	0.9989

$R^{2}$ is the indicator of fitting accuracy.

## Data Availability

Data will be made available on request.
